# Reviewing methodological approaches to dose-response modelling in complex interventions: insights and perspectives

**DOI:** 10.1186/s12874-025-02585-3

**Published:** 2025-05-16

**Authors:** Mollie Payne, Dominic Stringer, Ben Carter, Amy Hardy, Richard Emsley

**Affiliations:** 1 Department of Biostatistics and Health Informatics, Institute of Psychiatry, Psychology and Neuroscience, King’s College London, London, UK; 2 Department of Psychology, Institute of Psychiatry, Psychology and Neuroscience, King’s College London, London, UK; 3 South London and Maudsley NHS Foundation Trust, London, UK

**Keywords:** Dose-response, Complex interventions, Psychotherapy, Trials methodology, Systematic review

## Abstract

**Background:**

Understanding dose-response relationships is crucial in optimizing clinical outcomes, particularly in complex interventions such as psychotherapy. While dose-response research is common in pharmaceutical contexts, its application in complex interventions remains underexplored. This review examines existing statistical methods for modelling dose-response relationships in complex interventions, focusing on psychotherapy.

**Methods:**

A systematic literature search following PRISMA guidelines identified studies proposing novel statistical methods or innovative applications of methods for analysing dose-response relationships. The search encompassed various databases, yielding 224 articles. After screening and exclusion, seven studies were eligible for analysis. Data synthesis categorized methods into three groups: multilevel and longitudinal modelling, non-parametric regression, and causal inference with instrumental variables. Additionally, a survey was conducted among clinical researchers to understand their perspectives on dosing decisions in psychotherapy trials.

**Results:**

Multilevel and longitudinal modelling techniques, although informative, were only applicable to participants with sessional data, limiting causal interpretations. Non-parametric regression methods provided avenues for causal inference but were constrained by assumptions. Causal inference with instrumental variables showed promise in addressing these limitations, particularly in randomised controlled trials, yet still require a priori assumption of the dose-response function. The results of our survey suggested that there is not sufficient information available to clinical researchers to make empirical dosing decisions in psychotherapeutic complex interventions.

**Conclusions:**

This review highlights the scarcity of robust statistical methods for evaluating dose-response relationships in psychotherapy trials. The dose-response methodology applied to RCTs remains underdeveloped, hindering causal interpretations or requiring strong assumptions. Traditional approaches oversimplify outcomes, highlighting the need for more sophisticated methodologies. Clinical researchers emphasized the necessity for clearer guidelines and enhanced patient involvement in dosing decisions, echoing the broader findings of the review. Future research requires methodological advancements to inform effective decision-making in psychotherapy trials, ultimately optimizing patient care and outcomes.

**Supplementary Information:**

The online version contains supplementary material available at 10.1186/s12874-025-02585-3.

## Introduction

When considering dose-response, many researchers may automatically think of pharmaceutical contexts, where increasing doses of active drugs elicit a greater physiological response in the body. The consideration of dose-response in complex interventions, such as psychotherapy, may not be at the forefront of our minds. Despite this, understanding the effect of therapeutic dose on outcomes, in interventions such as psychotherapy or physiotherapy, is crucial to improving clinical outcomes for patients. The annual economic cost of mental health problems in the United Kingdom is estimated at £117.9 billion, with 44% of service providers reporting an inability to meet demands [1]. The need for optimal care is clearly demonstrated and a means of supporting this is by appropriately demonstrating optimal dose of treatment.

The issue of dose in psychotherapy was first discussed in 1986 by Howard et al., [2], defining one session of therapy to be analogous to a unit of medication. Throughout this paper, we will adopt this definition of dose in psychotherapy. In a meta-analysis of outpatient data Howard et al., [2] employed probit modelling to estimate how many sessions of psychotherapy were required for 50% of outpatients to reach an a-priori definition of clinical improvement. This analysis resulted in a log-linear dose curve, forming the foundations of the Dose Effect (DE) model in psychotherapy research. The DE model is defined by a negatively accelerating dose-response curve, indicating that while each therapy session provides benefit, the extent of this benefit diminishes with an increasing number of sessions.

A competing perspective is the Good Enough Level (GEL) model, proposed by Barkham et al., in 1996 [3]. This model is based on the findings from a randomised clinical trial (RCT) that compared the effect of 8 versus 16 sessions of psychotherapy. Using Jacobson and Truax’s (1991 [4]) criteria for clinically significant improvement (CSI), they analysed the proportions of patients that had improved at each session. They found a linear relationship between dose and response, with the strength of this relationship varying with total dose. The central argument of the GEL model is that the rate of change of improvement depends on the number of sessions of therapy, with those attending fewer sessions improving at a faster rate. The GEL suggests the presence of selection bias that occurs in complex interventions, where discontinuation rates are high, and the length of treatment may be intrinsically associated with the outcome. This situation is similar to flexible-dose pharmaceutical trials, where individuals requiring higher doses tend to show less improvement, leading to bias when comparing dose groups [5]. The key difference in the DE and GEL models lies in the conceptualisation of therapy duration; is it a facilitator of positive change, or is it dependent on the individual’s outcome? Research evaluating these models had produced equivocal evidence to date [6–9].

A systematic review of dose-response modelling methods in observational studies found that the majority of research utilises data from university counselling centres, targeting a specific demographic [10]. The statistical analyses used in these studies predominantly involved multilevel and longitudinal modelling, as well as comparisons of percentages. Most research in this field relies on routine care data. The lack of a comparator in this data means that causal interpretations cannot be made about the dose-effect curve. The gold standard for assessing effectiveness of dose is via RCT; therefore, methodologies applicable to clinical trials must be utilised.

Our primary aim is to identify all studies that propose a novel dose-response modelling method or apply an existing statistical method in a novel way to model dose-response, to complex intervention data, including observational, epidemiological and RCT data. This will facilitate a wider aim to understand the applicability of current methods to RCTs and the limitations of the statistical properties of these methods.

Our secondary aim is to clarify the process of determining dosage for emerging psychological interventions and to understand whether further empirical evidence is needed to inform these decisions. To achieve this, a survey was administered to gain insights from clinical researchers involved in the planning of complex interventions, in addition to our review of the literature.

## Methods

This review was conducted and reported in accordance with the most recent PRISMA guidelines [11]. The protocol was registered on PROSPERO on 12th May 2023 (CRD42023418381).

### Search strategy

A comprehensive literature search was conducted to identify papers focused on the novel development or application of statistical methodologies for analysing dose-response relationships in complex interventions. The search spanned five databases, including the Web of Science database and the OVID interface (encompassing MEDLINE, EMBASE, PubMed and PsychINFO databases). The search included all papers published from inception until 4th June 2023. Search terms were aligned with our main concepts of dose-response modelling, statistical methodology and complex interventions (See Supplementary Material A). Only papers published in English were included. Reverse referencing and forward citing techniques were employed to identify any additional relevant literature that might have been overlooked.

### Inclusion and exclusion criteria

Studies were included if they were (1) full-text articles, (2) written in English and (3) described novel statistical methods or novel applications of existing methods for estimating dose-response in complex interventions. Exclusion criteria comprised papers that (1) did not introduce new methods or applications, (2) reviews or meta-analyses, (3) protocols, (4) commentaries, (5) non-human studies, and (6) pharmaceutical studies.

### Screening

All search records were imported into Rayyan, a systematic review management tool [12]. Two independent reviewers screened the abstracts of identified studies, with any discrepancies resolved through discussion with a third reviewer.

### Data extraction and synthesis

The identified papers were synthesised based on the statistical methods presented. Narrative summaries were produced for each methodological group to provide an overview of the approaches utilised. The following data was extracted: (1) Year of publication, (2) Statistical method, (3) Study design, (4) Population, (5) Presence of control group, (6) Statistical Framework (Bayesian/Frequentist).

The identified papers were synthesised based on the statistical methods presented. Narrative summaries were developed for each methodological group to provide an overview of the approaches utilised.

### Survey for clinical researchers

A survey consisting of seven open-ended questions was administered using the Qualtrics platform (See Supplementary Material B). Minimal risk ethical approval was obtained from the author’s institutional ethics committee, and the survey was distributed via professional networks and social media channels. Responses were collected between June 2023 and December 2023.

## Results

### Search and study characteristics

**Fig. 1:**
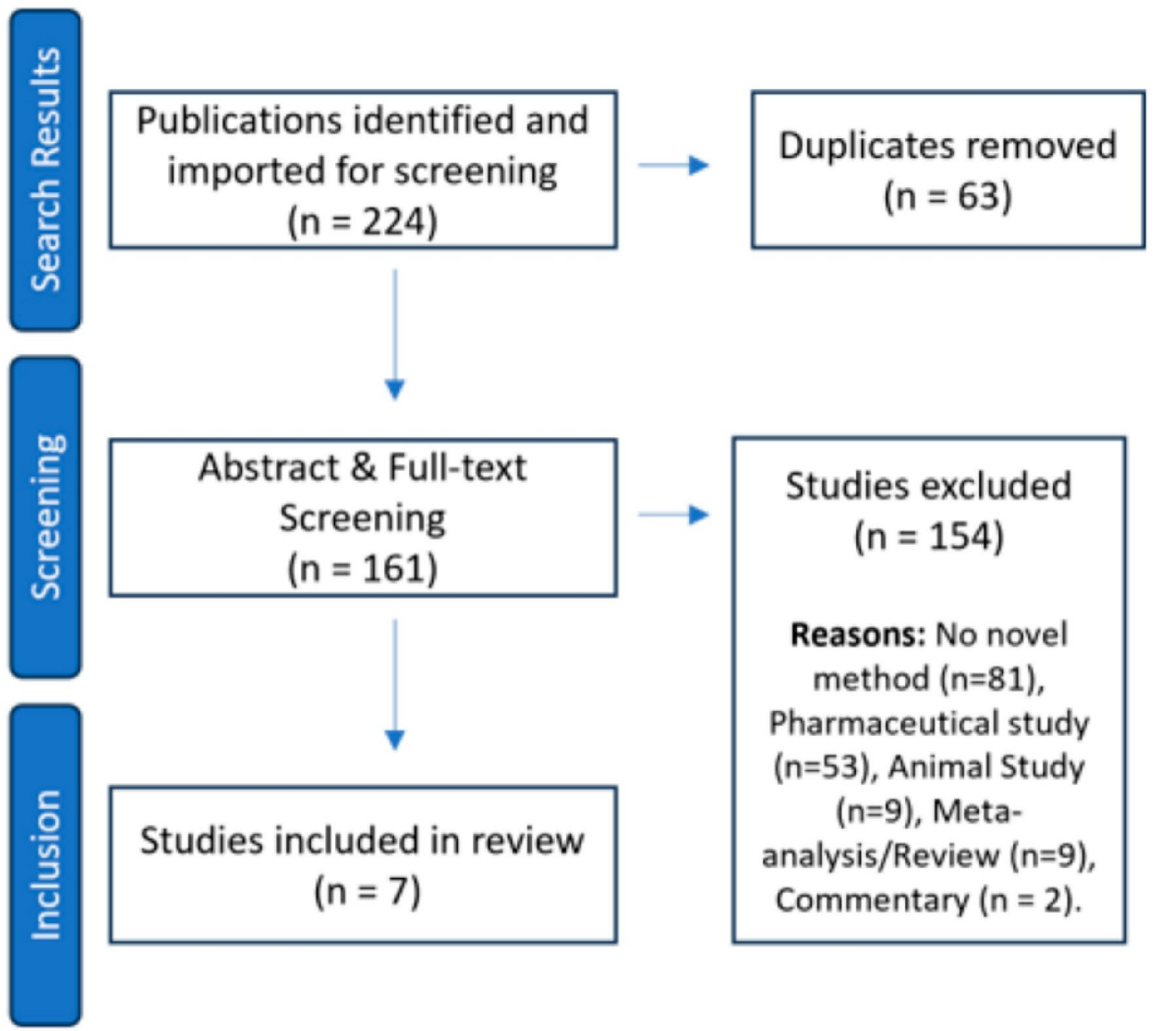
PRISMA Flow chart

Figure 1illustrates the number of studies included within each stage of the review process. Our search identified 224 peer-reviewed articles and abstracts, and 63 duplicates were removed. After applying the inclusion and exclusion criteria, seven studies were deemed eligible for full analysis (See Table 1). These studies were published between 1999 and 2017. Two studies utilised an observational design applied to an outpatient sample, three papers used an observational design without an application to data, two papers applied their methodology to RCTs with a parallel design in clinical samples. All identified studies employed a frequentist framework for their methods, and a comparative summary of each study’s methodological approach, strengths, limitations, and recommended applications is provided in Table 2.

**Table 1:** Characteristics of included methods

Authors	Year	Method	Study Design	Sample	Control arm
Lutz, Martinovich & Howard^13^	1999	Multilevel Modelling	Observational	Routine Care	No
Anderson & Lambert^14^	2001	Survival Analysis – Kaplan-Meier	Observational	Routine Care	No
Imbens^15^	2000	Propensity Score Matching	Observational	Not applicable	No
Steenland & Deddens^16^	2004	Smoothing Splines	Observational	Not applicable	No
Flores^17^	2007	Kernel Regression	Observational	Not applicable	No
Maracy & Dunn^18^	2011	Structural Mean Model + G-estimation	RCT	Clinical trial participants	Yes
Maracy & Dunn^18^	2011	2 stage Instrumental Variables	RCT	Clinical trial participants	Yes
Ginestet, Emsley & Landau^19^	2017	Instrumental Variables – Stein like estimators	RCT	Clinical trial participants	Yes

**Table 2:** Practical guide for how the methods identified can be used and the appropriate situations

Method	Research Question	Strengths	Weaknesses	Recommended uses
Mutli-level Modelling [13]	How many sessions are needed for 50%* of patients to reach CSI?	-Model captures heterogeneity through random coefficient -Inclusion of multiple characteristics potentially reduces unmeasured confounding	- Cannot make causal inferences regarding dose - Does not deal with selection bias or hidden confounding	This method is suitable for pilot studies aimed at identifying recommended treatment dose to test later in a RCT. All patients would receive treatment and the number of sessions they attend would be recorded. Researchers must collect detailed patient information to enhance the analysis.
Kaplan-Meier Curve [14]	How many sessions are needed for 50%* of patients to reach clinically significant improvement (CSI)?	- Allows for an exploration of relationships between characteristics and time to CSI -Suitable for analysing observable data without predefined time limits on therapy duration - Uses longitudinal, sessional data to model shape of response without relying on interpolation	- Cannot make causal inferences regarding dose - Sessional outcome data must be collected, which is time consuming - When a participant reaches CSI, this must be maintained until termination of treatment - Does not deal with selection bias or hidden confounding	This method is well-suited for pilot studies aimed at identifying recommended treatment dose to test later in a RCT. All patients would be offered treatment, their outcomes after each session must be collected using a valid psychometric measure. Average time to reach CSI data can be estimated for the sample. The resulting dose-response should be considered as a guideline, rather than an exact estimation of the dose-response relationship.
Smoothing Splines [16]	What is the potential shape of the dose-response curve?	-Allows for exploration of the potential underlying dose-response curve -Various shapes can be explored and compared using goodness-of-fit indices.	- Cannot make causal inferences regarding dose - The true dose-response curve can be oversimplified or overcomplicated -Goodness-of-fit indices may not reliably identify best more accurate model -Requires large sample size	This method is suited to large, observational studies where researchers want to explore the shape of the dose-response curve. Splines should be applied to observational data, goodness-of-fit indices will help to choose the most plausible shape of response. This is exploratory and should serve as a visualisation before adopting more formal models.
Propensity Score Method [15]	What is the average effect of dose conditional on propensity score?	- Allows for causal interpretation of the effect of dose - If assumptions are met, outcome is unconfounded by dose, given covariates. - Balances observed covariates between treatment levels	-Requires that outcome is unconfounded given observed variables - Subject to assumptions of overlap and positivity -Misspecification of outcome model leads to bias	This method is suited to observational studies where patients have self-selected varying doses of treatment. The method requires appropriate variables to be collected to create propensity score that balances the covariates that affect self-selection into dose levels.
Kernel Estimation [17]	What is the dose-response function of a continuous treatment?	- Does not require assumptions about the functional form of the dose-response - Allows for causal interpretation of the effect of dose - Balances observed covariates between treatment levels	-Requires that outcome is unconfounded given observed variables - Dependence on observable variables -Sensitive to sample size	This method is suited to large, observational studies where the relationship between covariates and treatment assignment is complex or poorly understood, such as when patients self-select into varying doses of treatment based on factors that interact in nonlinear or unknown ways.
SMM(G) IV(2SLS) IV(ATR)† [18]	What is the average treatment effect of the received dose, accounting for non-compliance.	- Allows for causal interpretation of the effect of dose - Possible to accommodate non-linear effects - Handles selection bias that arrives from self-selection into dose	- To identify randomisation as an instrument, we must assume no direct effect of randomisation on outcome (Exclusion restriction) - The functional form of dose must be pre-specified. - To model a non-linear effect, multiple valid instruments must be identified	These methods are best applied to randomised controlled trial data. The method estimates average treatment effects using variable session attendance. When modelling non-linear effects, it may be necessary to identify an additional instrumental variable.
Stein-Like Estimators [19]	What is the average treatment effect of the received dose, accounting for non-compliance.	- Allows for causal interpretation of the effect of dose - Combination of OLS and 2SLS reduces bias whist mitigating variance - Adapts to the strength of the instrument used - Handles selection bias that arrives from self-selection into dose	- To identify randomisation as an instrument, we must assume no direct effect of randomisation on outcome (Exclusion restriction) - Assumes homogeneity of treatment effects - Assumes linear dose-response relationship	These methods are best applied to randomised controlled trial data. The method estimates average treatment effects using variable session attendance. When modelling non-linear effects, it may be necessary to identify an additional instrumental variable. This method is suitable for when there is a concern between the bias and variance trade-off in standard IV methods.

Data synthesis revealed three distinct groups of methodology:

- **Multilevel and Longitudinal Modelling.** These included two methods that were based on naturalistic study designs using outpatient data [13, 14]. These methods required sessional data, which is only obtainable for those who receive treatment.
- **Non-Parametric Regression Methods.** These methods were based on observational and epidemiological study designs, where a clinical sample was not identified [15–17]. The methods did not accommodate a control group.
- **Causal Inference with Instrumental Variables (IV)**. These methods were specifically proposed for RCTs in mental health and included a control group in their methodology [18, 19].

### Data synthesis

### Multilevel and longitudinal modelling

We identified two papers that applied multilevel and longitudinal modelling to analyse dose-response data: a random coefficient model [13] and a Kaplan Meier survival model [14]. Both methods required sessional data, i.e., data collected in each session of therapy and therefore only available in the treatment arm, hence not allowing for a control comparator to be included.

Lutz et al., (1999 [13]) presented a random coefficient model to predict optimal dose for patients, based on vectors of their presenting characteristics (such as prior exposure to psychotherapy or therapist ratings of intake symptoms) and baseline outcome measures (Y_0_). The authors specify a model (Eq. 1) for the outcome at a specific session (Y(S)) as a linear function of the intercept (π_0i_), the slope (π _1i_), the logarithm of sessions (log(S)) and some random error (ε_is_).

1 $$Y\left( S \right)\, = \,{\pi _{0i}} + \,{\pi _{1i}}\left( {Log\left( S \right)} \right)\, + \,{\varepsilon _{is}}$$

The intercept (π_0i_) is estimated by a linear function of three values of subscales of the outcome during session one (X_1i_, X_2i_ & X_3i_). The slope (π_1i_) is estimated by a vector of seven presenting characteristics in a multilevel model, including a random slope (υ_1i_), which handles the heterogeneity that is not explained by the vector of characteristics. Using the average log change in outcome at each session, the number of sessions needed for 50% reliable improvement can then be calculated.

Anderson and Lambert (2001 [14]) demonstrated the application of survival analysis to estimate dose-response in psychotherapy by adapting parameters utilized in the Kaplan-Meier procedure. The Kaplan-Meier procedure is a non-parametric estimate of the survival function. Conventionally, the survival function is the probability of surviving past a specified time *(T = t)*. Death would typically be the event of interest. Each probability (*P*_*rt*_) is found by dividing the number of patients in who survived until *(t-1)* days and survived day *t*, by the number of patients alive at the end of day *(t-1).* We can estimate the probability of survival by calculating the product of the probability and conditional probabilities of surviving each value of *t* until *t-1*. In this application of survival analysis to dose-response, authors defined time (*t*) as the number of sessions received and replaced the event of interest with reaching clinically significant improvement (CSI). The model is used to estimate the cumulative probability of attaining CSI at each session.

### Non-parametric regression

We identified three papers that described non-parametric regression methods. These methods included model-based adjustment propensity score methods, a non-parametric kernel estimator, and the use of smoothing splines to visualise dose-response. These methods were applied to observational studies without control groups.

Imbens (2000 [15]) extended Rosenbaum and Rubin’s (1983 [20]) propensity score methodology to accommodate multiple treatment values, such as various doses. Primarily used in observational studies, where there are substantially varying covariates between groups, the covariates are balanced to correspond with the probability of belonging to a treatment group. The propensity score *(e(x))* is the probability of receiving treatment *(D = 1)*, given covariates (*X*). If treatment assignment is unconfounded given the pre-treatment covariates, then we adjust for *e(x)* in our model, rather than all pre-treatment covariates.

Alternatively, propensity scores can be used by weighting the inverse of the probability of receiving treatment. The weights are *1/e(X)* for those in the treatment group and *1/(1-e(X))* for those in the comparison group. These weights are estimated using a logistic regression model that predicts treatment. In this study, Imbens (2000 [15]) extended this model to incorporate multi-valued treatment by using a multinomial or ordered logit model to predict the probability of receiving one of the many treatments *(T = 1*,*…*,* k).* When we are interested in different levels of dose, Imbens (2000 [15]) suggests using a smoothing score in *T*. The final score estimates the conditional probability of receiving each treatment level given covariates, thus making treatment received independent of outcomes when conditioned on the score. Under the assumption of unconfoundedness, regression adjustment of the score allows for estimation of the causal effect of dose.

Flores (2007 [17]) proposed a non-parametric method for estimating the average dose-response function by identifying the location and size of optimal dose. Employing the potential outcomes approach [21], this method utilises non-parametric estimation techniques to remove restrictions on the functional relationship between dose and response. Similar to Imbens (2000 [15]), it is assumed that selection into dose is independent of outcomes, conditional on observable characteristics. The assumption of unconfoundedness is extended to a continuous treatment by assuming that selection into various levels of the treatment is made by observable covariates and unobserved variables are not associated with potential outcomes. This enables the estimation of the average dose-response function as a partial mean using kernel estimators.

Steenland and Deddens (2004 [16]) demonstrated the use of smoothing splines to visualize dose-response relationship in observational data. Splines are piece-wise polynomials defined by knots, that can be added to regression models to visualise curvature in outcome. Fewer knots create a smoother curve, whereas the curvature increases as additional knots are added. The authors present three approaches, including restricted cubic splines, penalised splines and LOESS plots. Potential shapes can be explored and compared using goodness-of-fit indices; however, the best fitting model may not always be the most appropriate model to use for understanding optimal dose.

Steenland and Deddens (2004 [16]) suggest visualising the dose-response curve prior to specifying a parametric model, but they warn to take caution on specifying the smoothness of the curve. For example, a smooth curve may reduce the impact of random noise but it may also overlook heterogeneity in the data.

### Causal inference with instrumental variables

Two studies described the use of causal inference methods using an instrumental variables (IV) approach. This method accounts for selection bias in the number of sessions attended and uses the outcome data from the comparator arm but can be statistically inefficient.

Maracy and Dunn (2011 [18]) discuss three methods for estimating the causal effect of dose: G-estimation for structural mean models (SMM(G)), 2-stage least squares (IV(2SLS)) and, adjusted treatment received (IV(ATR)). These methods share a common approach of using two-stage methods, based on the assumption that the treatment effect, given compliance and covariates in the treatment arm is equal the effect of average number of sessions.

In stage one, SMM(G) estimation involves regressing sessions on covariates for the treatment arm, to predict would be sessions for both arms (Ĉ). In stage two, the potential outcomes are estimated by regressing the outcome on covariates within each arm. The difference in potential outcomes (Δ) is calculated and regressed on predicted sessions (Ĉ). The method allows for quadratic modelling of the effect of dose by regressing the square of sessions on covariates to predict sessions squared (Ĉ^2^), which is then included in the stage two model.

Similarly for the IV(2SLS) method, sessions are regressed on randomised group and covariates. The outcome is then regressed on predicted sessions and covariates to estimate the average effect of psychotherapeutic sessions. For the IV(ATR) method, rather than predicted sessions being calculated, the residuals for sessions are calculated and used in stage two. Both methods allow for quadratic modelling of sessions, but the model would not be identified unless we had an additional variable that met the assumptions to be an instrument: a variable that is highly correlated with sessions but is not directly associated with the outcome.

Despite the slightly different processes involved in the use of G-estimation algorithms and IV regression, Maracy & Dunn (2011 [18]) demonstrated their equivalence in estimation if set-up appropriately, building on similar work in Dunn & Bentall (2007 [22]).

Ginestet, Emsley & Landau (2017 [19]) proposed combining the IV(2SLS) method with estimates from a linear dose model using Stein-like estimators. This methodology aims to address the trade-off between bias and variance in treatment effect estimation.

The outcome is then regressed on the Stein-like estimator and covariates to estimate the average treatment effect of dose. Stein-like estimators are calculated for each dose level by shrinking the 2SLS estimator towards the ordinary least squares (OLS) estimate. The combined estimate is weighted average of the 2SLS and OLS, with the weight determined by a tuning parameter α. The value of α is chosen to minimise the mean squared error, which in turn balances the unbiased IV estimates with the most efficient OLS estimates.

### Survey for clinical researchers

We collected qualitative survey data from seven clinical researchers, this included professors, readers and chief investigators. The average length of service in this field was 16.5 years (SD = 5.85). The qualitative survey feedback is summarised below.

### Insights in dosing decisions

The stakeholders involved in making decisions regarding dose in psychotherapy clinical trials primarily include the Principal Investigator (PI), psychological therapists and statisticians. The final decision-making role often lies with the PI or involves collaborative decision-making among stakeholders, informed by literature and consultations with clinical experts.

Key factors facilitating decisions surrounding dose include published papers, recommendations from clinical guidelines, expected effect sizes, practicality, cost, and previous successful studies. Conversely, factors inhibiting decision-making include funding constraints, lack of clear evidence or guidelines, and the balance between ideal and practical considerations.

### Perceptions of dosing decisions

Participants expressed a desire for clearer guidelines, improved patient and public involvement (PPI), and a more differential approach based on patient needs. They also emphasized the importance of planning dose decisions at the protocol/design stage of trials.

Generally, participants felt that there is insufficient information available to guide decision-making surrounding dose in the planning stage of psychotherapy clinical trials. They expressed interest in further research to address this gap and enhance decision-making processes.

## Discussion

**Fig. 2:**
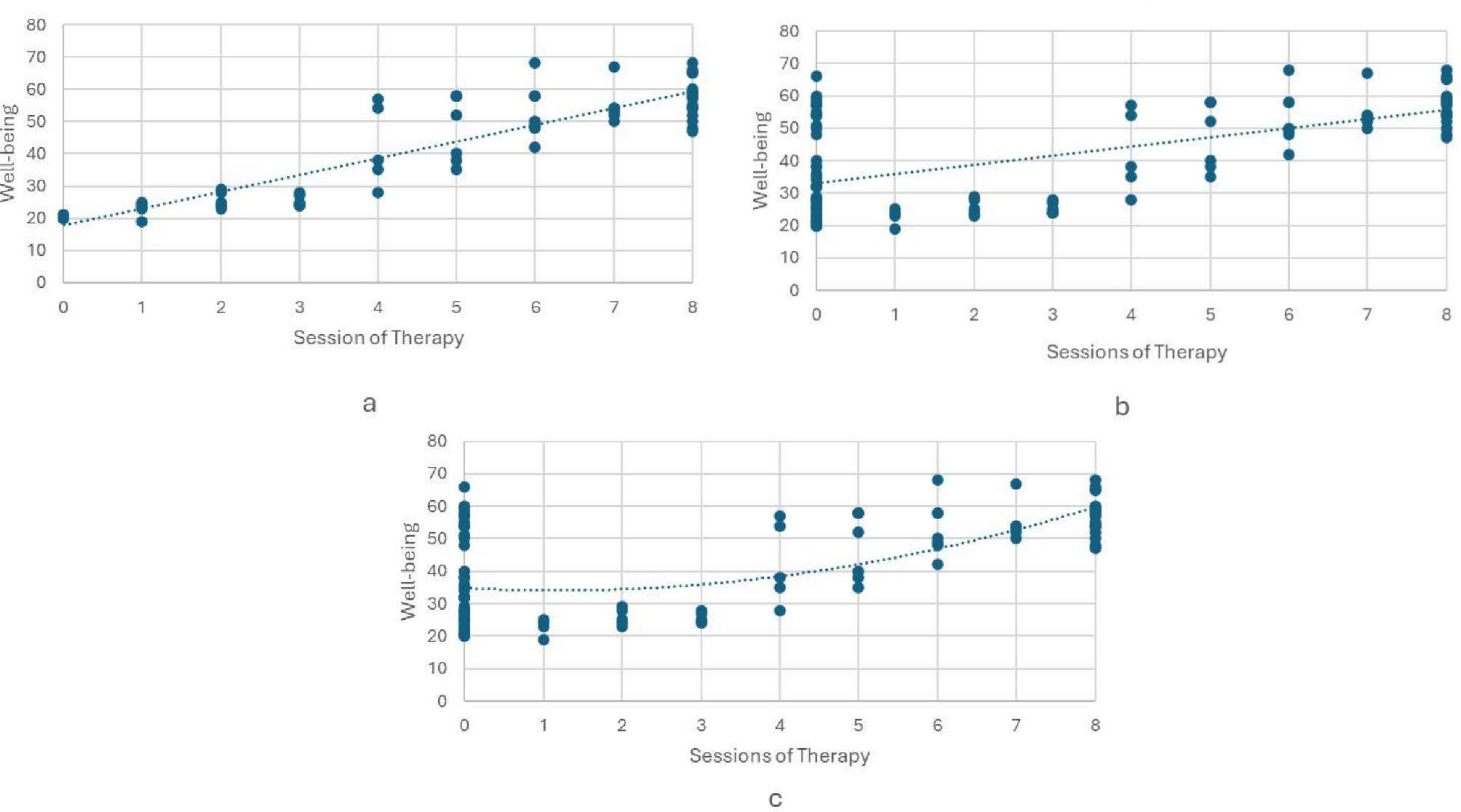
Conceptual comparison of dose-response modelling methods: (**a**) observational methods applied within treatment arm; (**b**) RCT-based methods allowing causal inference but assuming linearity; (**c**) RCT-based methods allowing both causal inference and non-linearity

This review aimed to identify statistical methods for evaluating dose-response relationships within complex interventions, focussing on psychological therapy. The methods identified are diverse, some allowing exploration of dose-response shapes, some that may inform future clinical trials and some that reduce hidden confounding in our estimates of the effect dose. Notably, a majority of the literature on dose-response modelling primarily utilised observational data, with only relatively recent application to RCTs. The methods developed for observational data, although able to model a multi-valued treatment, could in practice only be applied to the treatment arm in an RCT (see Fig. 2a)2, as multi-level modelling methods and spline methods require dose attended for the control arm which is often not defined. Propensity score methods, although theoretically could model outcome data from the control arm, the inclusion of the control arm in practice would violate the overlap assumption due to an excess of 0 session attendance; as the probability they would attend over 1 session, given covariates is 0. Methodologies developed for RCTs facilitate causal interpretation, as they compare the potential outcomes between arms (Fig. 2b). In theory, these methods could also model a non-linear dose-response (Fig. 2c) However, this would require prior assumption of the dose-response relationship and so far, has been only demonstrated with a quadratic effect of dose. 

Traditionally, dose-response research in psychotherapy has considered binary outcomes, “improved” versus “not improved”. This has allowed for the use of probit analysis to determine the dose required to achieve improvement in 50% of participants [2]. This framework has been adapted in multi-level modelling methods, such as Lutz et al., (2001 [13]), who estimated the number of sessions needed for improvement through linear interpolation. Similarly, Anderson and Lambert’s (2001 [14]) approach estimated time to improvement based on binary sessional outcomes. This dichotomous approach often overlooks the nuanced data collected in complex interventions, potentially missing critical aspects of mental health improvement.

A significant limitation of the multilevel and longitudinal methods is their requirement for sessional data, this makes them less applicable to a RCT setting as we cannot include the control arm. In the absence of a comparator, making causal inferences about dose effects becomes challenging.

Non-parametric regression methods offered a solution to the issue of causality by using the potential outcomes framework [15, 16, 21]. These methods allowed for causal interpretation from observational data, by controlling for the covariates in such a way that the dose received becomes unconfounded. Nonetheless, propensity score methods involve unblinded modelling of treatment effects to create scores, which can introduce bias in RCT settings. The choice of covariates in this model heavily influences the treatment effect, and substantial differences in covariate values can render results sensitive to assumptions of linearity and overlap. These concerns are highlighted in the causal inference literature [23, 24].

Among the few studies addressing dose-response modelling in psychotherapy RCTs, two publications employ causal inference instrumental variable methods [18, 19]. These methods estimate the potential outcomes under each arm, allowing for a causal interpretation of the effect of sessions on treatment effect. The instrumental variable approach tackles the issue of non-compliance by using randomisation as an instrument for measuring dose. Despite this, a major limitation to these approaches is the assumption of a linear relationship between session and treatment effect. This linearity assumption implies that each therapy session provides an equal benefit, which may not accurately reflect the varying impact of sessions over time or account for time-dependency between sessions [25]. Also, it doesn’t acknowledge that the content of sessions will vary across sessions and between individuals, unlike with pharmaceutical compounds which are constant over time and across the sample. Whilst it is possible to model a quadratic dose-response relationship using these methods, doing so requires specifying the functional form of the dose in advance and identifying a second instrument that satisfies stringent criteria for valid identification.

This highlights a further limitation of how dose is considered in psychotherapeutic context. The causal methods highlighted deal with dose as a function of non-adherence to treatment protocols, it is recognised that session engagement is not the optimal way to study dose [18]. The gold standard approach would be to conduct a clinical trial where patients are randomised to varying dose levels. However, in complex interventions this is not standard procedure, therefore these methods offer an alternative solution to understanding the effect of dose whilst mitigating selection bias.

We note that for all studies identified, dose was considered as a session of therapy. Unlike a pharmaceutical context, we can only infer the active dose of a psychological treatment, and this is not without measurement error [18]. As demonstrated in the review, we can use sessions as a proxy for dose, but this may not validly capture patient receipt of the active ingredients of the intervention. Instead, we could consider dose as the therapeutic targets that have been delivered, regardless of sessions. We could consider each session as a fractionated dose, representing a partial exposure to one dose. The debate regarding how to optimally define dose in psychological interventions is central to improving understanding of the impact of dose for any given treatment.

### Limitations and directions

A limitation of this review is that it does not encompass all methods used in dose-response studies of complex interventions. For example, Robinson et al. (2020 [10]) identified additional methods that are not covered here, such as simple regression and chi-square analysis. Their absence in this review is justified as these standard methods are so widely used that papers do not exist to explain their application to dose-response analysis. These methods are not consistent with the objective of this review, as they oversimplify the dose-response relationship by ignoring hidden confounding and selection bias (Maracy & Dunn, 2008 [18]). The work here demonstrates a clear gap in the literature for dose-response modelling methods, particularly of which use RCT data. Future work is needed to develop dose-response modelling methodology so that researchers are able to make valid causal inferences about the relationship between dose and response in psychotherapy interventions, without making pre-specifying the dose-response function. Without the correct methods available to model dose-response of complex interventions, our assumptions of the effect of psychotherapy thus far are potentially flawed. To optimise patient care, clinical trials need to be able to causally model dose-response in interventions to inform real world practice.

### Perspectives from clinical researchers

The secondary aim of this study was to gain deeper insights into clinical researchers’ perspectives regarding dose considerations in psychotherapeutic RCTs. Researchers identified factors influencing dosing decisions, including published literature, clinical guidelines, practicality, and cost considerations. Funding limitations and a lack of clear evidence pose challenges. Desired improvements include clearer guidelines, increased patient involvement, and tailored dosing approaches, ideally integrated during the trial design phase. Participants expressed a need for more comprehensive research to address these gaps and improve decision-making processes.

## Conclusions

This review highlights the limited availability of robust statistical methods for evaluating dose-response relationships in complex interventions, particularly within psychotherapy clinical trials. Whilst methods for observational data are well developed, RCT-specific methodology remains underdeveloped, hindering causal interpretations. Traditional approaches often oversimplify outcomes, failing to capture the complexity of mental health improvement. Although the causal inference IV methods attempt to address limitations, they are still constrained by assumptions about the dose-response function.

The findings of this review complement the results of our survey. Clinical researchers highlight the challenges surrounding dose decision-making in psychotherapy clinical trials, and the need for clearer guidelines, enhanced patient involvement, and a tailored approach to dosing. We emphasise the necessity for more sophisticated statistical methodologies in this area to inform effective decision-making in clinical trials.

## Electronic supplementary material

Below is the link to the electronic supplementary material.

Supplementary Material 1

Supplementary Material 2

## Data Availability

No datasets were generated or analysed during the current study.

## Abbreviations

DE: Dose Effect
GEL: Good Enough Level
RCT: Randomised Controlled Trial
CSI: Clinically significant improvement
IV: Instrumental Variables
SMM(G): Structural Mean Model with G-Estimation
IV(2SLS): Instrumental Variables with Two-Stage Least Squares
IV(ATR): Instrumental Variables with Adjusted Treatment Received
OLS: Ordinary Least Squares
PI: Principal Investigator
PPI: Patient Public Involvement
