# Supplementary material for: Reviewing methodological approaches to dose-response modelling in complex interventions: insights and perspectives
Source: BMC Med Res Methodol. 2025 May 16;25:135. doi: 10.1186/s12874-025-02585-3 (PMC12082932; doi:10.1186/s12874-025-02585-3)
Supplement: Supplementary file 1 — Supplementary Material 1 [file 12874_2025_2585_MOESM1_ESM.docx]

**Supplementary A - Search Strategy**

Web of Science database and the OVID interface from inception to present.

| **#** | **Searches** |
| --- | --- |
| 1 | dose?response OR dose response OR dose effect OR dose model OR dose response model* OR dose effect model* OR dose response effect* |
| 2 | statistic* OR method* OR model* OR estimat* OR mathematical model* OR propensity scor* OR instrumental variable* OR causal model* OR linear model* |
| 3 | psychotherap* OR counselling OR digital therap* OR complex intervention* OR mental health |
| 4 | 1 AND 2 AND 3 |
