# Supplementary material for: Reviewing methodological approaches to dose-response modelling in complex interventions: insights and perspectives
Source: BMC Med Res Methodol. 2025 May 16;25:135. doi: 10.1186/s12874-025-02585-3 (PMC12082932; doi:10.1186/s12874-025-02585-3)
Supplement: Supplementary file 2 — Supplementary Material 2 [file 12874_2025_2585_MOESM2_ESM.docx]

**Supplementary B – Survey Questions**

**Survey for Clinical researchers**

Understanding how decisions regarding dose of psychotherapy in clinical trials are made.

The following questionnaire seeks to understand how decisions regarding manualised dose of psychotherapy are made in clinical trials, including who makes these decisions and what factors may aid or constrain the decisions. Manualised dose is defined as the number and frequency of psychotherapy sessions, which is pre-specified in the protocol of a clinical trial. This involves all modalities of psychotherapy (for example, individual, group, in-person, digital).

1. In your team, who are the stakeholders that make decisions regarding dose?

2. What is your role in making these decisions?

3. What factors facilitate you and your team’s decisions surrounding dose?

4. What factors inhibit you and your team’s decisions surrounding dose?

5. What, if anything, would you like to see change in the decision making of dose in psychotherapy?

6. Do you believe that there is sufficient information available to guide decision making surrounding dose in a planning stage of a psychotherapy clinical trial?

7. If a trial design existed to optimise dose of psychotherapy, would you be interested in using this in your work?
